# Early Television Exposure and Children’s Behavioral and Social Outcomes at Age 30 Months

**DOI:** 10.2188/jea.JE20090179

**Published:** 2010-03-05

**Authors:** Shunyue Cheng, Tadahiko Maeda, Sakakihara Yoichi, Zentaro Yamagata, Kiyotaka Tomiwa, Japan Children’s Study Group

**Affiliations:** 1 Research Institute of Science and Technology for Society, Japan Science and Technology Agency, Tokyo, Japan; 2 The Institute of Statistical Mathematics, Research Organization of Information and Systems, Tokyo, Japan; 3 Department of Child Care and Education, Ochanomizu University, Tokyo, Japan; 4 Department of Health Sciences, School of Medicine, University of Yamanashi, Yamanashi, Japan; 5 Graduate School of Medicine, Kyoto University, Kyoto, Japan

**Keywords:** children, television viewing, behavioral outcomes, prosocial behavior

## Abstract

**Background:**

Previous research has suggested that television (TV) viewing may be associated with increased behavioral and emotional problems in children. However, there are few prospective studies targeted for its association with outcomes of children under 3 years old. The purpose of this study was to exam the association between children’s early TV exposure at ages 18 and 30 months and the behavioral and emotional outcomes at age 30 months.

**Methods:**

We analyzed data collected prospectively in the Japan Children’s Study. TV exposure was assessed by mothers’ report at infant ages of 18 and 30 months. The outcomes were assessed using the Strengths and Difficulties Questionnaire (SDQ). Analysis of Covariance was used to estimate the effect of TV exposure on behavioral and emotional outcomes.

**Results:**

The percentage of children who watched TV 4 hours or more per day was 29.4% at age 18 months, 24.5% at age 30 months, and 21% at both ages. Hyperactivity–inattention at age 30 months was positively associated with TV exposure at age 18 months, whereas prosocial behavior was negatively associated with hours of exposure even after adjustment. However, there were no significant differences in SDQ subscales according to daily hours of TV viewing at age 30 months.

**Conclusions:**

Daily TV exposure at age 18 months was associated with hyperactivity–inattention and prosocial behavior at age 30 months. However, the directly casual relation was not proved in the present study. Additional research considering the TV program content and exposure timing are needed to investigate the causal relation between TV viewing and behavioral outcome.

## INTRODUCTION

Many adverse effects of television (TV) viewing on children and adolescents have been documented, including obesity,^1^^,^^2^ aggressive behavior,^3^^–^^5^ decreased physical activity,^6^ behavioral problems,^7^^–^^9^ and sleep disorders.^10^^,^^11^

The effect of TV viewing on children’s developmental outcomes is likely to vary by age or neurodevelopmental stage of the child at the time of exposure, as well as by media content.^9^^,^^12^ The newborn brain continues to develop rapidly through the initial years of life and considerable plasticity exists during this period.^13^^,^^14^ Several studies have suggested that heavy TV exposure may have detrimental effects on behavior and social skills in early childhood, although the results, to date, have been mixed.^7^^,^^15^^,^^16^

The public health implications of early TV and video viewing are potentially great.^17^^–^^19^ There are theoretical and empirical reasons to believe that the effects of media exposure on children’s development are more likely to be adverse before the age of about 30 months than later in chldhood.^7^^,^^9^^,^^20^^,^^21^ Analyses have shown that TV viewing before age 3 years is associated with a deleterious effect on reading recognition and reading comprehension scores, with each additional hour/day leading to a reduction in scores of 0.31 and 0.58, respectively.^18^ By contrast, TV viewing at ages 3–5 years has been associated with an increase in the reading comprehension score of 0.51 per hour/day viewed.^18^ Thus, the effects of amounts of TV viewing on social development in infancy remain uncertain.

To our knowledge, few longitudinal studies have examined the association of TV viewing in infancy with behavioral and emotional development at ages younger than 3 years, and among them, only a few have examined the effect of sustained exposure over time or independent timing effects of exposure at different ages.^8^^,^^18^ With regard to the Japanese population, a few studies have examined the effects of TV viewing on infants’ social development.^22^^,^^23^ Kano et al^22^ reported negative effects of TV viewing on limited aspects of infant social development based on a cross-sectional study of infants at age 42 months. On the other hand, Sugawara^23^ reported no significant effects on three aspects of social and emotional development at infant age of 2 years based on three years of follow-up study. To our knowledge, the results are not necessarily consistent among previous studies on the Japanese population, and more evidence will be needed to confirm the effects of TV viewing on infants’ behavioral and emotional problems.

The purpose of this study was to test the hypothesis that early TV exposure at ages 18 and 30 months is associated with behavioral and emotional problems at age 30 months. In addition, to understand the differential impact of TV exposure by amount of exposure, we examined the association of sustained heavy TV viewing from ages 18 to 30 months with behavioral and emotional outcomes at age 30 months.

## METHODS

### Subjects

This study relied on data collected prospectively as part of the Japan Children’s Study (JCS) project. The JCS project is a prospective developmental cohort study started in 2005 by the JCS research group at three study sites: Osaka, Mie, and Tottori. The purpose of the project is to describe the development of sociability in children and to investigate factors affecting this development. The children’s parents completed a baseline questionnaire when the infants were 4 months old, to be followed up at ages 9, 18, and 30 months. The mother–child dyads also participated in laboratory observations at each measurement occasion. Participants were recruited at the three study sites mentioned above, and the recruitment procedure has been described in detail elsewhere.^24^ To summarize, all infants were born between August 2004 and April 2006. Mothers of nationality other than Japanese, families planning to move, and infants with serious medical complications were excluded. A total of 479 mothers with infants participated in the baseline assessment at infant age 4 months. Mothers completed the self-administered questionnaires sent to them before the 4-month laboratory observation. Of the 479 mother–child dyads, 361 (75%) mother–child dyads were followed until the children were 30 months old. They completed questionnaires at 4, 9, 18, and 30 months. Among them, 45 children were excluded because of missing data on TV exposure on one/more occasions; the study sample included 316 children whose mothers reported TV exposure at both 9 and 18 months. The dyads that did and did not complete the follow-up had similar demographic characteristics of baseline assessment, and no significant differences in distributions were found in the main study variables between the studied and excluded samples.

### Measures

*Dependent variables:* At 30 months, behavioral and emotional adjustment was assessed using the Japanese version of the Strengths and Difficulties Questionnaire (SDQ). The SDQ is a well-validated 25-item screening questionnaire composed of five scales that assess conduct and peer problems, hyperactivity–inattention, emotional symptoms, and prosocial behavior.^25^^–^^27^ Each item is rated as being not true (0), somewhat true (1), or certainly true (2), and each SDQ subscale consists of five items, thus yielding scores between 0 and 10. The first four problem scales are summed to generate a total difficulties score. The scores for the prosocial items are not incorporated (in the reverse direction) into the total difficulties score, as the absence of prosocial behaviors is conceptually different from the presence of psychological difficulties. The internal consistency (Cronbach’s alpha) of SDQ scales was 0.45 for emotional symptoms, 0.46 for conduct problems, 0.60 for hyperactivity–inattention, 0.52 for peer problems, and 0.74 for prosocial behavior. In our sample, Cronbach alpha values were low in some subscales, particularly for emotional and conduct problems, compared to those of Matsuishi.^26^ The reason will be discussed below.

*Independent variables:* Our primary predictor of interest was hours of TV watched per day at ages 18 and 30 months. In the surveys, the mothers were asked how much TV the child watched daily. The correlation coefficient between amount of TV viewing at 18 and 30 months was 0.54 (*P* < 0.0001). To examine the relationship between amounts of TV viewing and child outcomes by analysis of variance (ANOVA), the hours of TV viewing were categorized into four groups (<1, ≤1 to <3, ≤3 to <4, and ≥4). To assess the independent effects of TV viewing by the exposure time, viewing in each of 2 occasions was dichotomized according to whether the child watched fewer or more than 4 hours/day at each age. This process created 4 distinct categories of children according to their viewing histories: (1) watched fewer than 4 hours/day both at 18 and 30 months (low–low group); (2) watched fewer than 4 hours/day at 18 months, followed by more than 4 hours/day at 30 months (low–high group); (3) watched more than 4 hours/day at 18 months, followed by fewer than 4 hours/day at 30 months (high–low group); (4) watched more than 4 hours/day at both 18 and 30 months (high–high group).

Covariates: Several variables shown to be associated with the main dependent measures were included as covariates. These included the child’s birth weight (<2500 g or ≥2500 g), gestational birth age (<37 weeks or ≥37 weeks), child’s sex, number of children in family (only child or others), maternal educational level (≤high school or >high school), mother’s report of family income per year (<4 or ≥4 million yen), and level of mother’s cognitive stimulation (high or low). Information about maternal education, family income, and cognitive stimulation measured at 18 months was used in this analysis.

Measures of cognitive stimulation in the home were derived from items on the Environmental Evaluation Short Scale^28^ based on the Home Observation for Measurement of the Environment.^29^ For the youngest children, the cognitive stimulation score comprises 6 items related to mothers’ reading, telling stories, singing to their children, and playing with them. Five response categories reflected the frequency of participation (1 = never, 2 = once/twice a week, 3 = 3–4 times a week, 4 = 4–5 times a week, 5 = almost daily). The internal consistency of the scale in the JCS sample in our study was 0.60. We categorized these scores into two groups by the 75th percentile cut-off point: low (scores equal to or below the point) and high (scores above the point) of cognitive stimulation.

### Statistical analysis

Bivariate analysis explored the association between TV exposure and characteristics of the participants using Pearson chi-square statistics. ANOVA was used to evaluate the difference in scores of SDQ subscales on the different categories on TV viewing (four levels: <1, ≤1 to <3, ≤3 to <4, and ≥4). For subscales that showed significant difference in ANOVA, one-way ANCOVA was used to test whether the mean scores of each SDQ subscale still varied by daily hours of TV viewing at the ages of 18 and 30 months after adjustment for potential confounding variables, which included the child’s sex, birth weight, gestational age, number of children, maternal education, annual household income, and maternal stimulation level. Results are given as adjusted means with 95% confidence intervals (95% CI). ANCOVA was also used to examine the effect of TV viewing history on two occasions (four groups: low–low, low–high, high–low, high–high) on hyperactivity–inattention and prosocial behavior at 30 months. All analyses in this study were performed using SPSS Statistics 17.0.

### Ethics

The study protocol was approved by the Ethical Review Committee of collaborating research institutes (Osaka City General Hospital, Mie-chuo Medical Center, and Faculty of Regional Sciences, Tottori University) and the Ethical Review Committee of the Research Institute of Science and Technology for Society, Japan Science and Technology Agency, based on the “Guidelines Concerning Epidemiological Research” (Ministry of Education, Culture, Sports, Science and Technology and Ministry of Health, Labour and Welfare).

## RESULTS

A total of 302 mother–child dyads that completed all 4–30 month questionnaires on all relevant items were available for the following statistical analyses. Among the final study sample, 8.3% children had low birth weight, 3.6% were preterm children, 76.8% of the mothers had more than a high school education, and 68.9% had annual household incomes of 4 million yen or higher (Table 1). Approximately 29.4% children watched TV 4 hours or more per day at age 18 months, and 24.5% at age 30 months. With regard to TV viewing history, 21% children were in the high–high group, 7.6% in the high–low group, 12.5% in the low–high group, and 63% in the low–low group (Table 4). There was no sex difference in the amount of TV watching.

**Table 1.** Child and family characteristics and amount of TV viewing on each occasions (*n* = 302)

Characteristics	TV viewing at 18 months						TV viewing at 30 months				
	
	n (%)	<1	≧1 to <3	≧3 to <4	≧4	*P*^a^	<1	≧1 to <3	≧3 to <4	≧4	*P*^a^
Sex											
Male	153 (50.7)	10.5%	34.6%	22.9%	32.0%	ns	7.8%	47.1%	20.3%	24.8%	ns
Female	149 (49.3)	17.4%	37.6%	18.1%	26.8%		11.4%	49.0%	15.4%	24.2%	
Birth weight											
<2500 g	25 (8.3)	16.0%	40.0%	24.0%	20.0%	ns	8.0%	60.0%	12.0%	20.0%	ns
≥2500 g	277 (91.7)	13.7%	35.7%	20.2%	30.3%		9.7%	46.9%	18.4%	24.9%	
Gestational age											
<37 weeks	11 (3.6)	9.1%	27.3%	27.3%	36.4%	ns	.0%	45.5%	9.1%	45.5%	ns
≥37 weeks	291 (96.4)	14.1%	36.4%	20.3%	29.2%		10.0%	48.1%	18.2%	23.7%	
Number of Child											
Only child	166 (55)	11.5%	33.1%	24.8%	30.6%	ns	10.8%	44.6%	19.7%	24.8%	ns
Others	136 (45)	15.6%	38.3%	16.4%	29.7%		7.0%	51.6%	15.6%	25.8%	
Maternal education											
≤high school	70 (23.2)	18.8%	27.5%	18.8%	34.8%	ns	5.8%	40.6%	23.2%	30.4%	ns
>high school	232 (76.8)	12.7%	38.2%	21.1%	28.1%		11.0%	50.4%	15.8%	22.8%	
Family income (yen)											
<4 millions/year	94 (31.1)	16.3%	29.1%	16.3%	38.4%	0.065	3.5%	43.0%	18.6%	34.9%	0.026
≥4 millions/year	208 (68.9)	12.0%	39.1%	23.4%	25.5%		11.5%	50.5%	16.7%	21.4%	
Maternal stimulation											
Low	214 (70.9)	10.8%	37.3%	21.1%	30.9%	ns	11.3%	46.1%	16.2%	26.5%	ns
High	88 (29.1)	19.3%	36.1%	19.3%	25.3%		6.0%	55.4%	18.1%	20.5%	

^a^*P* values for Pearson chi-square test of difference in proportions.

In bivariate analysis, mothers of low income groups reported their children viewing more hours of TV, especially at age 30 months (*P* = 0.026). The other variables were not associated with the amount of TV viewing (Table 1). The mean scores of hyperactivity–Inattention, peer problems, and total difficulties were significantly higher in boys than in girls (Table 2).

**Table 2.** Means and standard deviation of strengths and difficulties scores

	Boys *n* = 153		Girls *n* = 149		*P*^a^
		
	Mean	SD	Mean	SD	
Emotianl Symptoms	2.02	1.67	1.88	1.77	0.273
Conduct Problems	3.17	2.06	3.16	1.77	0.689
Hyperactivity-Inattention	4.41	2.02	3.84	1.86	0.012
Peer-Problems	2.33	1.63	2.03	1.85	0.031
Prosocial Behavior^b^	5.04	2.41	5.47	2.52	0.191
Total Difficulties	11.9	4.08	10.9	3.98	0.016

^a^*P* values for a *t*-test of sex difference of means.

^b^High value are positive.

Table 3 presents the ANOVA results. In this unadjusted analysis, the mean score of hyperactivity–inattention was significantly higher with the increase of amount of TV viewing at the ages of 18 and 30 months, whereas mean scores of prosocial behavior decreased with increasing hours of TV viewing only at the age of 18 months. The *P* values for the subsequent linear trend analysis for all these associations are also shown in Table 3. There were no significant differences among categories of watching TV at any of two occasions on emotional symptoms and conduct and peer problems.

**Table 3.** Mean score differences of Strengths and Difficulties Questonnaires (SDQ) according to daily hours of TV viewing

Television viewing per day	TV viewing at 18 months					TV viewing at 30 months				
	
	Mean	95% CI	*F*	*P*^b^	*P*^c^	Mean	95% CI	*F*	*P*^b^	*P*^c^
Emotianl Symptoms			0.62	0.599	ns			0.42	0.739	ns
<1	1.83	(1.4–2.3)				2.03	(1.4–2.6)			
≧1 to <3	2.03	(1.7–2.4)				2.04	(1.8–2.3)			
≧3 to <4	2.11	(1.7–2.6)				1.81	(1.3–2.3)			
≧4	1.79	(1.4–2.2)				1.82	(1.4–2.3)			
Conduct Problems			0.08	0.973	ns			0.89	0.449	ns
<1	3.07	(2.5–3.7)				2.86	(2.1–3.6)			
≧1 to <3	3.17	(2.8–3.5)				3.19	(2.9–3.5)			
≧3 to <4	3.11	(2.6–3.6)				3.44	(2.8–4.1)			
≧4	3.22	(2.8–3.7)				3.00	(2.6–3.4)			
Hyperactivity-Inattention			6.21	<0.0001	<0.0001			2.99	0.031	0.004
<1	3.26	(2.7–3.8)				3.31	(2.6–4.1)			
≧1 to <3	3.83	(3.5–4.2)				3.99	(3.7–4.3)			
≧3 to <4	4.45	(3.9–4.9)				4.28	(3.8–4.8)			
≧4	4.61	(4.3–5.0)				4.53	(4.1–4.9)			
Peer-Problems			0.27	0.844	ns			1.08	0.359	ns
<1	2.14	(1.6–2.7)				2.31	(1.7–2.9)			
≧1 to <3	2.17	(1.9–2.5)				2.09	(1.8–2.4)			
≧3 to <4	2.08	(1.7–2.5)				2.50	(1.9–3.1)			
≧4	2.28	(1.9–2.7)				2.08	(1.7–2.4)			
Prosocial Behavior^a^			3.34	0.020	0.004			1.07	0.361	ns
<1	5.98	(5.2–6.7)				5.71	(4.7–6.6)			
≧1 to <3	5.42	(5.0–5.9)				5.39	(5.0–5.8)			
≧3 to <4	5.26	(4.7–5.8)				4.88	(4.2–5.4)			
≧4	4.65	(4.1–5.2)				5.08	(4.6–5.7)			

^a^High value are positive.

^b^*P* value for omnibus *F* test of group mean differences.

^c^*P* value for a linear trend for means.

Table 4 presents the ANCOVA results. The hyperactivity–inattention problems remained significantly associated with the amount of TV viewing at age 18 months (the mean score increased linearly from 3.41 to 4.59) even after adjusting for potential confounders. On the other hand, the association between hours of watching TV and hyperactivity–inattention problems at age 30 months disappeared after adjustment. For the prosocial problems, there was no effect of TV exposure at any occasion, but there was a significantly linear trend (trend *P* = 0.039); the scores of prosocial behavior decreased (from 5.80 to 4.73) with increasing amount of TV watching at age of 18 months.

**Table 4.** Adjusteda scores of hyperactivety-inattention, prosocial behavior according to the daily hours of TV viewing at 18 and 30 months

TV viewing (hour/day)	*n* (%)	Hyperactivity-Inattention					Prosocial Behavior				
	
		Mean	95% CI	*F*	*P*^b^	*P*^c^	Mean	95% CI	*F*	*P*^b^	*P*^c^
At 18 months				3.73	0.012	0.002			1.89	0.132	0.039
<1	42 (14.0)	3.41	(2.7–4.1)				5.80	(4.9–6.7)			
≧1 to <3	109 (36.1)	3.81	(3.4–4.2)				5.50	(4.9–6.0)			
≧3 to <4	62 (20.5)	4.26	(3.7–4.8)				5.21	(4.5–5.9)			
≧4	89 (29.4)	4.59	(4.1–5.0)				4.73	(4.2–5.3)			
At 30 months				1.47	0.224	0.071			.45	0.718	0.352
<1	29 (9.6)	3.64	(2.8–4.4)				5.56	(4.3–6.5)			
≧1 to <3	145 (48.0)	3.94	(3.6–4.3)				5.36	(4.9–5.8)			
≧3 to <4	54 (17.8)	4.18	(3.5–4.7)				4.96	(4.0–5.6)			
≧4	74 (24.5)	4.48	(4.0–5.0)				5.09	(4.6–5.8)			
At both occasions				3.03	0.029				2.20	0.088	
High-high group	51 (21.0)	4.49	(3.9–5.1)				5.00	(4.3–5.7)			
High-low group	23 (7.6)	4.70	(4.0–5.4)				4.32	(3.4–5.2)			
Low-high group	38 (12.5)	4.40	(3.5–5.3)				5.31	(4.2–6.4)			
Low-low group	190 (63)	3.79	(3.5–4.1)				5.48	(5.1–5.8)			

^a^Adjusted child sex, birth weight, gestational age, birth order, maternal education and family income and maternal stimulation.

^b^*P* value for omnibus *F* test of group mean differences.

^c^*P* value for a linear trend for means.

The results for the effect of TV viewing history were as follows: The effect of TV viewing history was significant for hyperactivity–inattention, where children categorized to high–high and high–low groups showed higher mean scores compared to the low–low (reference) group. On the other hand, the effect of this variable on prosocial behavior was somewhat ambiguous, since the omnibus F test was marginally significant (*P* = 0.088), and if the post-hoc comparison of means is to be conducted with this sample, only the mean for high–low group was shown to be significantly lower than the low–low group.

## DISCUSSION

We found that early TV exposure was associated with subsequent mental health problems, especially those of hyperactivity–inattention and prosocial behavior. Approximately 1 in 3 children aged 18 months, 1 in 4 children aged 30 months, and 1 in 5 children at ages 18 and 30 months viewed 4 hours or more of TV per day. The percentage of children watching TV 4 hours or more at the age of 18 months in our study is higher than another previous study of Japanese children,^30^ where 12% of children fall into this category.

Our analysis provides a replication and enhancement of previous studies of the association between early TV exposure and behavioral problems and social skills.^7^^,^^16^^,^^21^ The present results are consistent with a previous study that found a significant association between TV exposure in very early (at age 1) and subsequent attention problems,^7^ and also with a previous study that found a significant association between TV exposure at 18 months and poor social development at the age of 3.6 years.^30^

In addition, our results offer evidence regarding the importance of exposure timing. The findings suggest that hyperactivity–inattention and prosocial problems are associated with hours of daily TV viewing at 18 months but not associated with hours of TV viewing at 30 months. After adjusting for the possible confounders, children in the group with the low–low viewing pattern were less likely to show hyperactivity–inattention problems than children having the high–high and high–low viewing patterns, which confirms the effect of amount of TV exposure. Children in the low–low group showed the highest score on prosocial skills, with the effect supported only by the post-hoc mean comparison after marginal significance in the omnibus F test. In total, the concurrent effect of TV exposure at 30 months of age was not confirmed on any subscale of SDQ. However, it is not clear why the children in the high–low group showed highest mean score in hyperactivity–inattention and lowest in prosocial behavior. It is possible that viewing TV before the age of 2 years has was more strongly associated with behavior and social skills at 30 months than viewing TV on later occasions, for example, between 2 and 3 years of age. Christakis et al^7^ reported that hours of TV viewing per day at ages 1 and 3 were associated with attention problems at age 7. Our findings concerning children at 18 months of age are consistent with the previous study that reported the adverse effect of TV exposure before age 2 years on subsequent child behavior outcomes; however, we did not find that effect of TV exposure at 30 months. The differences in study design may account for the different results. For example, our study measured TV exposure at both 18 and 30 months, and the outcomes were evaluated at age 30 months, thus examining partially the concurrent effect of the exposure, whereas the previous study measured TV exposure at 1 and 3 years and outcomes at age 7 years. The effect of TV exposure may not be strong enough to appear in the children’s concurrent behavior in our study because the period of exposure is too short. Continued efforts on outcome evaluation by a follow-up study will be necessary to measure the impact of exposure at that age. Another plausible explanation for the different results is that the TV content the children watched was different by age, or if the content was the same, the acquisition of language skills could have moderated the adverse effect of the amount of TV exposure. A recent study found that viewing educational TV programs before age 3 was not associated with attention problems 5 years later. In contrast, viewing of either violent or non-violent entertainment programs before age 3 was significantly associated with subsequent attentional problems.^9^ Our results may be interpreted from the perspective that children older than 2 years were more influenced by TV content than children younger than 2 years, because the older children have acquired more skills in comprehensive language, whereas very young children are more directly influenced by the amount of TV exposure than by the content.

Several limitations to this study warrant consideration. First, we relied on maternal reports on amount of TV viewed. Although this may not be an entirely accurate measure of the true amount, there are no a priori reasons to believe its imprecision would bias our findings in one direction or another. Second, we had no data on the content of the programs viewed. Besides, we do not have any information on how infants and their caregiver usually watch TV programs. For example, whether the infants are left alone in front of the TV or whether they watched together with their mother and some interaction between them may have occurred was not tested in our study. In other words, “quality” of TV watching was not measured; this constitutes another limitation of our study. Some research indicates that educational programs may in fact promote attention and reading skill among school-aged children,^17^ while others have posited that even educational programs can be detrimental.^31^ If exposure to certain kinds of programs is beneficial, even at a very young age, then the proportion of the program content may have moderated the detrimental aspects of other programs and should have moved the results toward the null point. The reality is that we have found a significant effect, which implies that the amount of TV watching has indeed at least some effect. However, more research is needed on the effects of the varying content of the TV watched, particularly for children of preschool age. Third, because the SDQ scale that we used for the assessment of behavior and emotional problems was intended for children aged 4–12 years,^26^ it does not necessarily indicate accurately the status of problems for children in the age range of our study. Additionally, parents have difficulty in rating a child’s emotional and behavior problems because of the considerable amount of normative opposition among toddlers in this age group as compared with higher age groups; a possible reason why the Cronbach alpha values for emotional and conduct problems was lower. Fourth, there may be additional contextual factors that influence behavioral and social skill outcomes. Although we adjusted for several potential confounders, there may be additional confounders including parental depression^32^^,^^33^ that were not captured in our study.

Despite these limitations, our study had several strengths. First, we examined the effect of TV exposure on several aspects of children’s emotional and behavioral outcomes. Although many studies have examined the effects of TV viewing on subsequent attentional problems,^7^^,^^9^^,^^15^^,^^16^ few studies have explored the association between different amounts of TV viewing and various aspects of emotional and behavioral problems.^8^ Our study emphasizes the need to perform longitudinal studies with different aspects of mental health as outcomes when studying the relationship between TV exposure and mental health. Second, these findings provide information on the association between exposure before age 2 with later behavioral outcomes may be stronger than TV exposure after age 2.

Several studies have reported the effect of media exposure on children younger than 3 years. However, among these studies, few examined separately the impact of TV exposure on children younger than 2 years and those older.^7^ Finally, our results also provide evidence regarding the importance of exposure time; sustained heavy exposure (our high–high group) are associated with both hyperactivity–inattention problems and poor social skills (compared with sustained less exposure; low–low group) but is necessarily considered to have highest association (in comparison with the high–low group). Further understanding of these relationships requires an increased focus on the content of media exposure.

In conclusion, our results enrich the understanding of the association of early TV exposure with subsequent behavioral and social development of children. Our findings suggest that the amount of infant TV viewing before 2 years of age is more strongly associated with hyperactivity–inattention problems at 30 months than that after 2 years of age. However, the significant association found in the present study did not necessarily prove the direct causal relation between the TV exposure and subsequent behavioral outcomes. It is possible that the strong association between the amount of TV exposure and later behavioral outcomes might be derived from reduced active interaction between the children and their caregivers as indicated by a current study.^34^ Other theoretically possible explanations for the association is, for example the overall attitude of caregivers who allowed their children to watch TV longer was the major contributing factors for the behavioral outcomes as suggested by one of the previous literature^11^ should also be taken into account. Furthermore, in view of the inconsistent results on the effects that have been reported in the previous studies in Japan, we would recommend collecting further information on the same aspects of children’s emotional and behavioral problems. Future research would need to consider the exposure time, program content, and quality of interactions between the infant and caregiver during TV viewing sessions.
